# Characteristics and outcome of acute myeloid leukemia with uncommon retinoic acid receptor-alpha (*RARA*) fusion variants

**DOI:** 10.1038/s41408-021-00561-w

**Published:** 2021-10-16

**Authors:** Laura Cicconi, Anna Maria Testi, Pau Montesinos, Eduardo Rego, Hong Hu Zhu, Hiroyuki Takahashi, Michael Dworzak, Elihu Estey, Anthony Schwarer, Jordi Esteve, Ombretta Annibali, Roberto Castelli, Milena Mirabile, Mario Angelini, Vladimir Lazarevic, Jeevan Kumar, Giuseppe Avvisati, Carmelo Gurnari, Franco Locatelli, Maria Teresa Voso, Miguel Angel Sanz, Francesco Lo-Coco, Oussama Abla

**Affiliations:** 1 Department of Biomedicine and Prevention, University Tor Vergata, Rome, Italy; 2 UOSD Ematologia, ASL Roma 1, Rome, Italy; 3 Department of Translational and Precision Medicine and Hematology, ‘Sapienza’ University, Rome, Italy; 4 Department of Hematology, Hospital Universitari i Politècnico la Fe, València, Spain; 5 Department of Internal Medicine, Medical School of Ribeirao Preto, Ribeirao Preto, Brazil; 6 Department of Hematology, the First Affiliated Hospital, College of Medicine, Zhejiang University, Hangzhou, China; 7 Department of Pediatrics, Toho University, Tokyo, Japan; 8 Children’s Cancer Research Institute and St Anna Children’s Hospital, Department of Pediatrics, Medical University of Vienna, Vienna, Austria; 9 Clinical Research Division, Fred Hutchinson Cancer Research Center, Seattle, WA USA; 10 Department of Hematology and Oncology, Eastern School, Monash University, Melbourne, VIC Australia; 11 Servicio de Hematología, Instituto Clínic de Enfermedades Hematológicas y Oncológicas, Hospital Clínic de Barcelona, Barcelona, Spain; 12 Hematology and Stem Cells Transplantation Unit, University Campus Bio-Medico, Rome, Italy; 13 Department of Biomedical and Clinical Sciences Luigi Sacco, University of Milan, Luigi Sacco Hospital, Milano, Italy; 14 HematologyUnit, Ospedale di Civitanova Marche, Macerata, Italy; 15 Hematology Unit, Ospedale Mazzoni, Ascoli Piceno, Italy; 16 Department of Hematology, Oncology and Radiation Physics, Skåne University Hospital, Lund, Sweden; 17 Department of Clinical Haematology and BMT Tata Medical Center, Kolkata, India; 18 Department of Pediatric Hematology/Oncology, IRCCS Ospedale Pediatrico Bambino Gesu, Rome, Italy; 19 Department of Pediatrics, Sapienza, University of Rome, Rome, Italy; 20 Division of Hematology/Oncology, Department of Pediatrics, the Hospital for Sick Children, University of Toronto, Toronto, ON Canada

**Keywords:** Translational research, Acute myeloid leukaemia

**Dear Editor,**

Acute promyelocytic leukemia (APL) is characterized by the reciprocal balanced translocation t(15;17)(q24.1;q21.2), which fuses the promyelocytic leukemia (*PML*) and retinoic acid receptor-α (*RARA*) genes, responsible for inhibition of cellular differentiation and proliferation of undifferentiated elements [1]. *RARA* can be fused to partners other than *PML* in rare cases of acute myeloid leukemia (AML), presenting with features suggestive of APL. At least 14 variant translocations have been identified, including *ZBTB16-RARA* [2], the most frequent one, followed by *NPM-RARA* [2], *NuMA-RARA* [3], *STAT5B-RARA* [3], *PRKAR1A-RARA* [3], *BCOR-RARA* [3], *FIP1L1-RARA* [3], *OBFC2A-RARA* [4], *GTF2I-RARA* [5], and the more recent *IRF2BP2-RARA* [6], *FNDC3B-RARA* [7], *TBLR1*/*RARA* [8], *STAT3/RARA* [9], and *NUP98*/*RARA* [10].

These cases display limited sensitivity to molecularly targeted therapies active in *PML-RARA*-positive APL, with the majority (i.e., *ZTBTB16-RARA*, *STAT5B-RARA*) showing in vitro and/or in vivo resistance to all-*trans* retinoic acid (ATRA) and arsenic trioxide (ATO) [1, 2].

Given their rarity, data on the clinical characteristics and outcomes of these patients are limited to small case series with the largest including 11 *ZBBTB16-RARA* and 2 *NPM1-RARA* leukemias [1]. Herein we report the clinico-biological features, treatment strategies, and outcomes of 24 cases of AML with rare *RARA* rearrangements diagnosed between 2005 and 2017, collected from 16 study groups/institutions in Europe (Italy, Spain, Austria, Sweden), China, Brazil, Japan, India, Australia, and United States. Common case report forms were sent to all participating centers and laboratory data including flow cytometry, cytogenetics, and molecular biology were centrally reviewed. Four cases had been previously reported [11–13]. The study was approved by the research ethics boards of all participating centers. Thirty-eight consecutive *PML-RARA*-positive APLs diagnosed at the Policlinico Tor Vergata University in Rome from 2005 and 2019 were selected as controls. Statistical analyses were performed using the SPSS software and the distributions of time-to-event variables were estimated by the Kaplan–Meier method. As to therapy outcomes, complete remission (CR), partial remission (PR), molecular remission, and primary refractory disease were defined according to the European Leukemia-Net AML guidelines [14]. Induction death was defined as occurring after the start of therapy and before achievement of CR while overall survival (OS) and event-free survival (EFS) were calculated from diagnosis until death and until failure to enter CR, relapse, second malignancy, or death, respectively. Twenty-four patients with a median age of 47 years (range, 2–83 years) were included in this study, with 4 pediatric patients (age range, 2–17 years). Three cases (12.5%) were classified as therapy-related AML: UPN 8 and 12 underwent previous radiotherapy for seminoma and breast cancer, respectively, while UPN 6 had a history of rheumatoid arthritis treated with hydroxychloroquine and methotrexate. Complete clinical and biological data of the 24 AML *RARA* variants are presented in Supplementary Table 1. At presentation, median white blood cell (WBC) and platelet counts were 11.5 × 10^9^/L (range, 1.83–248) and 75 × 10^9^/L (range, 8–229), respectively, compared to WBC 1.5 × 10^9^/L (range, 0.39–113,240) and platelets 25.5 × 10^9^/L (range, 7–218) in the 38 cases of PML-RARA+ve APL (*p*: 0.004, *p*: 0.00049). Eight patients (40%) presented with laboratory signs of coagulopathy but the levels of fibrinogen were higher as compared to PML-RARA+ve APL levels (Fib. 176 vs 121; *p*: 0.007). Comparison of clinico-biological data between *RARA* variants and PML-RARA+ve APL cases is reported in Table 1.

**Table 1:** Comparison of clinico-biological characteristics at diagnosis of *RARA* variants and PML-RARA-positive APL.

	*RARA* variants	APL	*p* value
Age, years (range)	47 (2–83	51.5 (14–82)	0.2
Sex F/M	5/19	15/23	0.1
Morphology M3/M3v	23/1	33/5	0.5
WBC, ×10^9^/L (range)	11.5 × 10^9^/L	1.58 × 10^9^/L	0.04
Platelets, ×10^9^/L (range)	75.5 × 10^9^/L	25.5 × 10^9^/L	0.0009
Fibrinogen, mg/dL (range)	176 (60–675)	121 (62–237)	0.007
FLT3-ITD (POS/NEG)		5/8	
Early death	2/24	5/38	0.6
Outcome of induction (CR)	16/24	33/38	0.1
EFS, 24 months/48 months, %	54.1/34	70.3/62.9	0.2
OS, 24 months/48 months, %	72/60	86.7/82.9	0.07

Morphologic features of leukemic blasts were compatible with hypergranular M3-AML in all cases except UPN 21 resembling a variant form of APL. Flow cytometry characterization was conducted using FACS calibur flow cytometer, with CD33, CD34, CD117, and HLA-DR always assessed. Data were available in 20/24 (83%) cases and showed strong positivity for CD33, negativity for CD34 and HLA-DR in all but 1 case in which CD34 was expressed at low level. Leukemia blasts of 8 patients (40%) expressed CD56. As compared to 20 control cases of *PML-RARA-*positive APL, the expression of surface markers of *RARA* variants and APL cases was identical, except for CD56 antigen that was more frequently and strongly expressed in AML with *RARA* variants (Fig. 1). Figure 1 shows the morphological and flow cytometry data of *RARA* variants. As to cytogenetic studies, chromosome banding was performed using standard techniques, and karyotypes were described according to the International System for Human Cytogenetic Nomenclature. Fluorescence in situ hybridization (FISH) was conducted with commercial probes: (i) *LSI RARA* dual-color break apart, (ii) *PML-RARA* dual-color dual fusion, or (iii) specific BAC probes for rare translocation partners [7]. Molecular studies to verify the presence of *ZBTB16-RARA* [2], *STAT5B-RARA*, *NuMA-RARA* [2], *PRKAR1A-RARA*, and *FIP1L1-RARA* were performed according to the published methods [3, 12]. Eighteen cases (75%) harbored ZBTB16-RARA, 3 (12.5%) STAT5B-RARA, and 1 each PRKAR1A-RARA, NuMA-RARA, and FIP1L1-RARA rearrangements. Additional chromosomal abnormalities were reported in four cases of ZBTB16-RARA and in two cases of STAT5B-RARA. In all cases, the presence of ZBTB16-RARA and STAT5B-RARA was confirmed by reverse transcriptase polymerase chain reaction. In the PRK1RA-RARA, NuMA-RARA, and FIP1L1-RARA cases, FISH analysis using a break apart probe for *RARA* was necessary to show the presence of the rearrangement.

**Fig. 1:**
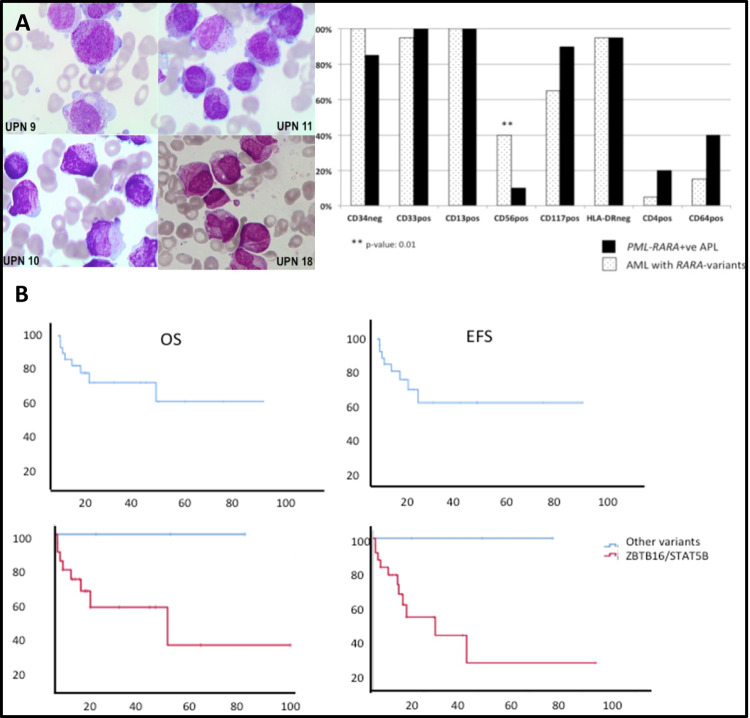
Biologic characteristics and survival outcomes of leukemias harboring RARA-variants. **A** Morphologic features of *ZBTB16-RARA* variant and flow cytometry characteristics of AML with RARA variants and PML-RARA-positive APL. The majority of blasts have hypergranular cytoplasm; in a minority of cases, Auer rods were present (UPN10 and UPN18). The majority of blasts harbored a regular round or oval nucleus that was more suggestive of FAB type M2; however, a minority of blasts in UPN 11 and UPN 18 showed irregular and indented nuclei. **B** Survival outcome of patients with *RARA* variant translocations.

Regarding induction therapy, 14/24 patients (58%) received ATRA together with AML-like induction, including cytarabine (ARA-C) and anthracyclines (daunorubicin) or idarubicin (IDA). Seven patients received APL-like induction therapy: ATO+ATRA+/−chemotherapy in 4 and ATRA plus anthracyclines in 3. One of the three elderly patients considered unfit for intensive therapy received low-dose ARA-C (L-DAC) and ATRA, a second received hydroxyurea (HU) plus ATRA, and the third received ATRA alone.

Sixteen of the 24 (67%) patients achieved hematologic CR (12 after a first and 4 after second induction). Two patients died early due to acute renal failure and bacterial pneumonia, respectively. PR was observed in 3 patients, including the two treated with L-DAC/ATRA and HU/ATRA, while 3 patients (13.6%) showed resistant disease. All three received re-induction with chemotherapy (*n* = 2) or ATRA/ATO (*n* = 1) but all died due to progressive disease (*n* = 2) and infection (*n* = 1). No hemorrhagic or thrombotic events were recorded during induction.

Eighteen out of 22 patients (82%) proceeded to consolidation; 13 received intensive chemotherapy with (*n* = 7) or without ATRA (*n* = 5) and one with gemtuzumab ozogamicin (GO). Two additional patients were consolidated with GO (alone or with ATRA,). Two elderly patients received multiple cycles of ATRA and L-DAC, while 1 younger patient received azacytidine as consolidation and was in stable disease (SD) at 7 months from diagnosis.

Seven patients underwent allogeneic hematopoietic stem cell transplant (allo-HSCT) in first CR: 4 from an HLA-identical sibling, 2 from matched unrelated, and 1 from a haplo-identical donor, respectively. At the time of analysis, 6 were alive and leukemia-free at a median follow-up of 25 months (range, 9–86), and 1 died from transplant-related toxicity. With a median follow-up of 11.5 months (range, 1–86), OS in our cohort (24 patients) at 12 months was 67% (confidence interval (CI) 95%, 46.1–87.8) and EFS was 64.1% (CI 95%, 41.8–86.4) (Fig. 1). At the time of analysis, 13 patients are alive in CR (54%), 3 are alive in PR/SD (12.5%), and 8 died (33.3%) due to disease progression (*n* = 5), transplant-related complications (*n* = 1), and early during induction (*n* = 2). Four patients (17%) relapsed during follow-up (3 *ZBTB16-RARA* and 1 *STAT5b-RARA*) at a median of 24 months (range: 10–36).

When analyzing separately the outcomes of ATRA-resistant genetic variants (*ZBTB16-RARA* and *STAT5B-RARA*), OS and EFS at 12 months were 60.9 and 56.7%, respectively (Fig. 1B). In contrast, all three cases with *PRKAR1A-*RARA, *NuMA-*RARA, and *FIP1L1-RARA* rearrangement are long-term survivors at 15–69 months from diagnosis; all three were treated with ATRA/CHT and without HSCT.

When comparing the outcome of *RARA* variants with that of 38 PML-RARA+ve APL treated with AIDA (*n* = 20) or ATRA-ATO (*n* = 14), OS and EFS after a median follow-up of 60 months were superior in the APL cohort but were not statistically different. The major cause of death in the cohort of APL was early death (*n* = 5, 13%) due to thrombo/hemorrhagic complications (Table 1). There was no significant statistical (*p* < 0.05) correlation between survival, age, secondary AML status, additional cytogenetic abnormalities and receipt of HSCT, recognizing that the ability to detect such correlations was limited by small numbers.

To our knowledge, our study represents the largest reported clinical series of adult and pediatric AML with *RARA* variant translocation. As shown here, this entity presents with strong morphologic and immunophenotypic resemblance to APL. CD56 has been previously reported to be expressed in AML cases harboring *RARA* fusion variants [14] and we herein confirm that CD56 was more frequently expressed in RARA variants as compared to *PML-RARA*-positive APL.

In our series, WBC, platelet counts, and fibrinogen levels were significantly lower in *PML-RARA*-positive APL as compared to *RARA* variants. Further, despite the presence of hypofibrinogenemia in 40% of patients with *RARA* variants, no thrombo-hemorrhagic events were reported, while early death due to hemorrhagic events in our control APL group was 13%. In the clinical practice, suspicion of a case of *RARA* variant leukemia should always raise in cases of strong morphologic and immunophenotypic (CD33, CD13 positive, HLA-DR negative, and CD56 positive) resemblance with APL but with negative results for PML-RARA fusion gene/transcript. Specific *RARA* break apart probe should be employed in those highly suspect cases.

Most importantly, although 23 of our 24 cases received ATRA in the present series, differentiation with ATRA and/or ATO was not evident in any of the patients with either *ZBTB16-RARA* or *STAT5B-RARA* rearrangements.

Indeed, *ZBTB16-RARA* and *STAT5B-RARA* variants display poor response to retinoids both in vitro and in vivo but few studies have showed some degree of differentiation after ATRA and/or cytostatic drugs [2, 11, 15]. Survival exceeding 12 months was achieved in two elderly patients with L-DAC or HU plus ATRA, which might represent a therapeutic option for frail patients.

Considering younger patients in the current series (<60 years), the choice of therapy in almost all cases consisted of an AML-like induction therapy. Previous series of *ZBTB16-RARA* and *STAT5B-RARA* cases have indeed consistently shown low CR rates and dismal survival outcomes despite intensive approaches [2, 11, 15], in sharp contrast to the very low relapse rate reported in *PML-RARA*-positive APL treated with ATRA-ATO [1]. Refractory/relapsed disease occurred in 30% of the cases of our series, with previous studies reporting 45–80% relapse rate in STAT5B and ZBTB16-RARA patients [2, 11]. The outcomes of the four pediatric patients included in our series and treated with intensive approaches seem to follow the trend of the younger adult patients.

Although the optimal post-consolidation therapy remains to be determined, in light of the available evidence [2, 11], most of our patients with ATRA-resistant variants (*ZBTB16-RARA* and *STATB-RARA*) were allocated to transplant-based approaches. When analyzing the survival outcomes of our series in comparison with the 38 control APL cases, there was a trend toward a superior OS in the APL group with EFS that was superior but not statistically different. In the interpretation of this data, several data must be taken into account: (i) the much longer follow-up of the APL cohort (60 vs 12 months); (2) the early death rate (13%) in the APL group caused by thrombo-hemorrhagic events; and (3) higher mortality due to relapse, resistant disease, and transplant-related complications in ATRA-resistant variants.

Our study is limited by the small number of patients, the heterogeneous molecular subtypes, different age groups, and the presence of secondary AML cases. Nevertheless, it represents the first large series that describes in detail the clinical and biological features of AML patients with these uncommon *RARA* variants. Despite the phenotypic and morphologic similarities with *PML-RARA*-positive APL, these *RARA* genetic variants show important clinical and biological diversity that accounts for resistance to molecularly targeted therapies and worse outcomes when considering the intensity of the approaches.

Next-generation sequencing studies are ongoing to unravel possible molecular differences at the basis of the sharply different biological and clinical behavior.

## Supplementary information

Table 1 Supplementary
