# Supplementary material for: Characteristics and outcome of acute myeloid leukemia with uncommon retinoic acid receptor-alpha (RARA) fusion variants
Source: Blood Cancer J. 2021 Oct 16;11(10):167. doi: 10.1038/s41408-021-00561-w (PMC8520532; doi:10.1038/s41408-021-00561-w)
Supplement: Supplementary file 1 — Table 1 Supplementary [file 41408_2021_561_MOESM1_ESM.docx]

**Table 1 Supplementary. Clinical and biological characteristics of *RARA*-variants**

**Table 1. Supplementary Legend.**

*CR, complete remission; **mCR, molecular complete remission; +Res, resistant disease; °ED, early death; §PR, partial remission; ATRA, All-trans retinoic acid; ATO, arsenic trioxide; ARA- C, cytarabine; DNR, daunorubicin; DA, daunorubicin and ARA-C; ETO, etoposide; FLA-GO, fludarabine, cytarabine, gemtuzumab ozogamycin; GO, gemtuzumab ozogamycin; HU, hydroxyurea; HiDAC, high-dose cytarabine; IDA, idarubicin; ICE, idarubicin, cytarabine, etoposide; L- DAC, low-dose cytarabine; MTZ, mitoxantrone; MTX, methotrexate.

HSCT, hematopoietic stem cell transplantation; MUD, matched unrelated donor

| **UPN** | **Age** | **Sex** | **Flow cytometry** | **Morphology** | **WBC** | **Plts** | **Fibrinog en** | **Karyotype** | **Genetics** | **Induction** | **Consolidation** | **HSCT in 1^st^ CR** | **Relapse** | **Outcome** (months after diagnosis) |
| --- | --- | --- | --- | --- | --- | --- | --- | --- | --- | --- | --- | --- | --- | --- |
| 1 | 74 | M | CD13+,CD33+,MPO+, CD34-,CD56-  ,HLA-DR-, CD117- | M3 | 5.3 | 93.0 | 180  mg/dL | 47,XY,+22 [5] /46,XY[30] | PRKAR1A-RARA | ATRA+IDA+ATO, **CR*** | ATRA+ARAC+Amsacri ne | None | None | Alive in CR1, 15m |
| 2 | 17 | M | MPP+CD13+,CD33+, HLA-DR-,CD34- | M3 | 2.1 | 170.0 | 84mg/dl | 46, XY, der(13), der(17q)[20] | STAT5B/RARA | ATRA+AML-BFM 04 ,  **CR*** | AML-BFM 04 | None | 2  relapses (24 mos) | Dead, 41 m |
| 3 | 56 | M | NA | M3 | - | - | NA | 46,XY,t(11;17)(q23;q21)[20] | ZBTB16-RARA | ARAC+IDA, **CR*** | GO | None | None | Alive in CR1, 24m |
| 4 | 14 | M | CD33+,CD13+,CD117+,MPO+,CD56+, HLA-DR, CD34- | M3 | 20.0 | 138.0 | 85mg/dl | 46,XY,t(11;17)(q23;q21)[18] | ZBTB16-RARA | ATRA+ICE, **mCR**** | ATRA+GO | **HLA-id sibling** | None | Alive in CR1, 86m |
| 5 | 52 | M | NA | M3 | 7.83 | 94.0 | 89mg/dl | 46,XY,t(11;17)(q23;q21)[20] | ZBTB16-RARA | ATRA+ARA-C+DNR,  **Res;** 2nd IND:IDA+ ARA-C+GO,**CR** | IDA+ ARA-C+GO | **MUD** | None | Dead for TRM, 9m |
| 6 | 50 | F | CD33+,CD13+,CD117+, CD56+,CD34-  ,HLA-DR- | M3 | 2.9 | 26.0 | 312mg/dl | 46,XX,del(5)(q13q31),t(11;17)(q23;q21)[2  0], 46,XX[10] | ZBTB16-RARA | ATRA+ICE, **CR*** | ATRA+MTZ+ ARA-C  (2 cycles) | **HLA-id sibling** | None | Alive in CR1, 36m |
| 7 | 67 | M | CD33+,CD13+,CD117+, CD56+,CD34-  ,HLA-DR- | M3 | 53.0 | 69.0 | 67mg/dl | 46, XY, t(11;17) (q23;q21)[20] | ZBTB16-RARA | ATRA+IDA, **CR*** | AIDA (high-risk) | None | None | Alive in CR1, 8m |
| 8 | 33 | M | NA | M3 | 248.0 | 29.0 | 187mg/dl | 46, XY, t(11;17) (q23;q21)[12] | ZBTB16-RARA | ATRA+DNR+ARA-C,  **CR*** | ATRA+DNR+ARA-C | **HLA-id sibling** | 3  relapses (24 mos) | Alive in CR4, 53m |
| 9 | 75 | F | CD33+, CD117+, CD13+, HLA-DR-  ,CD34- | M3 | 44.0 | 64.0 | 154mg/dl | 46, XY, t(11;17) (q23;q21)[12] | ZBTB16-RARA | ATRA,**ED°** | - | - | - | Dead, 1m |
| 10 | 83 | M | CD45+, CD33+, CD117+, CD13+, CD4+, CD56+, MPO+, CD34-, HLA-DR- | M3 | 6.0 | 34.0 | 338mg/dl | 46, XY, t(11;17) (q23;q21)[20] | ZBTB16-RARA | ATRA+HU **PR*** | ATRA+L-DAC | None | None | Alive in PR, 12m |
| 11 | 41 | M | CD33+, CD13+, MPO+, CD117+, HLA- DR-, CD34-, CD56- | M3 | 9.67 | 96.0 | 60mg/dl | 46, XY, t(11;17) (q23;q21)[12] | ZBTB16-RARA | ATRA+IDA+ARA-C,  **CR*** | ATRA+HiDAC (2  cycles) | **MUD** | None | Alive in CR1, 11m |
| 12 | 77 | F | NA | M3 | 8.0 | - | NA | 46, XY, t(11;17) (q23;q21)[18] | ZBTB16-RARA | ATRA+DNR+ARA-C,  **CR*** | DNR+ARA-C (2  cycles) | None | 1 relapse  (36 mos) | Alive in CR2, 36m |
| 13 | 38 | M | CD13+CD33+CD45+CD56+CD117+CD 34-HLAD-DR- | M3 | 10.1 | 65.0 | 344  mg/dL | No metaphases | ZBTB16-RARA | ATRA+DNR, **CR*** | 1stHDAC+DNR; 2^nd^ HDAc | **HLA-id sibling** | None | Alive in CR1, 9 m |
| 14 | 38 | F | CD33+, CD13+,CD117+, CD34+/-, HLA-DR-, CD56+/- | M3 | 23.6 | 43.0 | 664 mg/dl | 46,XX,t(11,17)(q23;q22)[20] | ZBTB16-RARA | ATRA+DNR+ARA-C,  **Res;** 2^nd^ IND: MTZ  +ARAC+ETO,Res§ | - | None | - | Dead, 3m |
| 15 | 48 | M | CD33+,CD13+, CD117+, HLA-DR, CD34-, CD56- | M3 | 71.0 | 41.0 | 675mg/dl | No metaphases | ZBTB16-RARA | ATRA+DNR, **Res;** 2^nd^ IND: ATO+HiDAC,  **Res^§^** | - | None | - | Dead,6m |
| 16 | 28 | M | CD33+, CD13+, MPO+, CD34-, HLA- DR-, CD56- | M3 | 23.8 | 113 | NA | 46, XY, t(11;17) (q23;q21)[13]/45, idem,- Y[10]/46,XY[3] | ZBTB16-RARA | ATRA+DNR+ARA-C,  **PR*** | 2^nd^ line:AZA,stable disease | None | - | Alive with stable disease, 7 m |
| 17 | 36 | M | CD33+, CD13+, CD117+,MPO+, CD34-  , HLA-DR-, CD56- | M3 | 11.5 | 40.0 | NA | 46, XY, t(11;17) (q23;q21)[10]/46,del(11)(q14) [3]/46,XY[7] | ZBTB16-RARA | DNR+ARA-C, **PR*;**  2^nd^ ind HiDAC**, CR** | 2 HiDAC | None | 1 relapse  (10 mos) | Alive in PR, 11 m |
| 18 | 76 | M | CD33+,CD13+, CD117+, MPO+,CD56+, CD34-, HLA-DR- | M3 | 44.0 | 140.0 | 171mg/dL | 46, XY, t(11;17) (q23;q21) [15] | ZBTB16-RARA | L-DAC+ATRA, **PR** | L-DAC+ATRA | None | - | Dead in PR, 12 m |
| 19 | 3 | M | MPO+,CD33+,CD13+, CD34-, HLADR-  , CD56- | M3 | 29.6 | 139.0 | 247  mg/dL | 46,XY,del(1)(p?),add(7)(q32),del(11)(q?),-  14,-16,-16,-17,-22, +mar1,  +mar2,+mar3,+mar4,+mar5 [13/20]/46, XY, add(21),+mar5,+mar6[4/20], 46, XY[3/20] | NuMa-RARA | ATRA+ARA-C+DNR,  **CR** | ATRA+ARA-C+MTX  (4 cycles) | None | None | Alive in CR, 42 m |
| 20 | 47 | M | CD45+CD33+CD13+CD64+CD117dim HLA-DRdimCD56-CD34- | M3 | 12.3 | 82.0 | 107  mg/dL | 46,XY,t(11,17)(q23;q21)[6]; 46, XY[6] | ZBTB16-RARA | ATRA+ATO (RIF),  **Res**; 2^nd^ induct ARAC- IDA, **CR** | HDAC (1 cycle); MAE (1 cycle); DA (1 cycle) | **Haplo SCT** | None | Alive in CR, 13 months |
| 21 | 59 | M | CD45+CD33+CD13+CD64+CC117dim CD34-CD56-HLA-DR- | M3v | 7.7 | 8.0 | 387  mg/dL | 45, X0,-Y[30] | ZBTB16-RARA | ATRA+ATO,**Res** | - | None | - | Dead, 3 months |
| 22 | 2 | F | MPO+CD13+CD15+Cd65+CD34- HLADR-CD34-CD56- | M3 | 34.0 | 61.0 | 545  mg/dL | 46,XX, t(4;17)(q11;q12) [20/20] | FIP1L1-RARA | ATRA+ARA-C+DNR,  **CR** | ATRA+ARA-C+MTX  (4 cycles) | None | None | Alive in CR, 69m |
| 23 | 47 | M | MPO+CD13+CD33+CD56+CD34- HLADR- | M3 | 1.6 | 123 | 122  mg/dL | 46,XY,der(5)t(5;8)(q31;q13)[11]/46,XY[3] | STAT5B-RARA | ATRA+ARA-C+DNR,  **Res**; ATRA+FLAG- IDA, **CR** | - | - | None | Alive in CR, 3 m |
| 24 | 34 | M | CD13+CD33+MPO+CD117+CD123+C D34-HLADR- | M3 | 1.83 | 131.0 | 56 mg/dL | 46, XY [20] | STAT5B-RARA | ATRA, ARA-C, IDA, ATO, **ED°** | - | - | - | Dead, 1 m |
